# Sepsis-associated thrombotic microangiopathy–like syndrome following cardiac surgery: a case report

**DOI:** 10.3389/fcvm.2026.1838488

**Published:** 2026-09-18

**Authors:** Chujun Yang, Shanshan Dong, Zhijing Xia, Wan Peng, Hua Yan, Zhenlu Zhang, Qimei Wei

**Affiliations:** 1 Department of Clinical Laboratory, Wuhan Asia Heart Hospital, Wuhan University of Science and Technology, Wuhan, China; 2 School of Medicine, Department of Medicine, Wuhan University of Science and Technology, Wuhan, China; 3 Department of Cardiology, Wuhan Asia Heart Hospital, Wuhan University of Science and Technology, Wuhan, China

**Keywords:** ADAMTS13, cardiac surgery, sepsis, sepsis-induced coagulopathy, thrombotic microangiopathy-like syndrome, von Willebrand factor

## Abstract

Severe sepsis following cardiac surgery may be accompanied by a thrombotic microangiopathy (TMA)-like syndrome distinct from classic immune-mediated thrombotic thrombocytopenic purpura (TTP) and overt disseminated intravascular coagulation (DIC). We report a 62-year-old man who developed sepsis after mitral valve replacement, complicated by thrombocytopenia (nadir 45 × 10⁹/L), microangiopathic hemolysis (2.6% schistocytes), and multiple organ dysfunction. Laboratory findings revealed marked dysregulation of the von Willebrand factor (vWF)/ADAMTS13 axis, with markedly elevated vWF antigen levels (548%), moderately reduced ADAMTS13 activity (32.8%), and negative ADAMTS13 inhibitors. Despite meeting criteria for sepsis-induced coagulopathy (SIC), the patient did not fulfill diagnostic criteria for overt DIC, as fibrinogen levels remained elevated and D-dimer elevation was limited. The patient improved with comprehensive sepsis management and supportive care, including adjunctive plasma infusion. Marked vWF/ADAMTS13 imbalance with preserved fibrinogen and limited D-dimer elevation may help distinguish sepsis-associated TMA-like syndrome from overt DIC.

## Introduction

Thrombotic microangiopathy (TMA) encompasses a spectrum of disorders characterized by thrombocytopenia, microangiopathic hemolytic anemia, and organ dysfunction. Classic immune-mediated thrombotic thrombocytopenic purpura (TTP) represents a well-defined entity caused by severe ADAMTS13 deficiency, typically <10%, in association with inhibitory autoantibodies (1). In contrast, critically ill patients with infection, trauma, or major surgery may develop TMA-like manifestations driven primarily by endothelial activation and systemic inflammation rather than autoimmune ADAMTS13 inhibition (2, 3).

Sepsis is increasingly recognized as a state of immunothrombosis and endotheliopathy, in which massive release of von Willebrand factor (vWF) from activated endothelial cells overwhelms relatively preserved ADAMTS13 activity, promoting platelet-rich microthrombus formation (4, 5). This process has previously been described as a “TTP-like syndrome” and has also been referred to as sepsis-associated thrombotic microangiopathy in recent literature, although it remains pathophysiologically distinct from classic TTP and overlaps with other sepsis-related coagulopathies (2, 6).

Patients undergoing cardiac surgery are particularly vulnerable due to cardiopulmonary bypass–induced endothelial injury, systemic inflammation, and postoperative infection. However, diagnostic differentiation between sepsis-associated TMA-like syndrome, sepsis-induced coagulopathy (SIC), and disseminated intravascular coagulation (DIC) remains challenging, and misclassification may lead to inappropriate management. Here, we present a case of sepsis-associated TMA-like syndrome that developed in the setting of postoperative sepsis following mitral valve replacement, highlighting the diagnostic reasoning and therapeutic considerations.

## Case presentation

A 62-year-old male presented on May 8, 2025, with intermittent exertional chest pain relieved by rest. He had a history of mitral valve prolapse with regurgitation, previously managed with diuretics and inotropic therapy. He had no history of hypertension, diabetes, or dyslipidemia. On admission, he was alert, with a heart rate of 120 bpm, respiratory rate of 21/min, and blood pressure of 123/78 mmHg. Lung auscultation was clear, cardiac rhythm was irregular without murmurs, the abdomen was soft, and there was no peripheral edema. Transthoracic echocardiography revealed posterior mitral leaflet and posterolateral commissure prolapse with chordae tendineae rupture, severe mitral and tricuspid regurgitation, and a left ventricular ejection fraction (LVEF) of 55%. Cardiac computed tomography angiography confirmed posterior mitral leaflet prolapse with annular calcification. Electrocardiography demonstrated atrial flutter and frequent ventricular premature complexes. Initial laboratory testing showed thrombocytopenia (platelets 93 × 10⁹/L), elevated D-dimer (2.26 µg/mL), mildly elevated high-sensitivity cardiac troponin I (0.0238 ng/mL), and elevated NT-proBNP (3,077 pg/mL). Liver and renal function tests and urinalysis were unremarkable. The patient was diagnosed with valvular heart disease with severe mitral regurgitation, non-rheumatic tricuspid regurgitation, atrial flutter, and frequent ventricular premature complexes.

On May 13, the patient underwent bioprosthetic mitral valve replacement, radiofrequency ablation maze procedure, left atrial appendage closure, and tricuspid annuloplasty under deep hypothermic cardiopulmonary bypass (duration 4 h 15 min). The early postoperative course was stable, with minimal pericardial drainage (120 mL/24 h). On postoperative day 2 (POD2), the patient acutely developed high-grade fever (maximum temperature 39.3 °C), hypoxemia (SpO₂ 85%), and acute respiratory distress syndrome. Central venous pressure was 2–3 mmHg, arterial blood gas showed metabolic acidosis (pH 7.18), and oliguria was accompanied by a 95 µmol/L increase in serum creatinine within 24 h. Repeat echocardiography revealed global hypokinesis with a reduced LVEF of 32%, consistent with septic cardiomyopathy.

Laboratory evaluation demonstrated leukopenia (white blood cell count decreased from 14.6 to 3.3 × 10⁹/L), progressive thrombocytopenia (from 111 to 74 × 10⁹/L), anemia (hemoglobin decreased from 142 to 123 g/L), elevated lactate dehydrogenase (629 U/L), and schistocytes accounting for 2.6% of red blood cells on peripheral smear, indicating microangiopathic hemolytic anemia. Inflammatory markers were markedly elevated, including interleukin-6 (38 103 pg/mL) and procalcitonin (47.28 ng/mL). Coagulation testing revealed prolonged prothrombin time (PT 22.3 s), a declining D-dimer level (1.29 µg/mL), and elevated fibrinogen concentrations (4.18 g/L), suggesting preserved or increased fibrinogen synthesis rather than consumptive coagulopathy. Given the combination of thrombocytopenia and microangiopathic hemolysis in the setting of severe sepsis, sepsis-associated TMA-like syndrome was suspected. ADAMTS13 activity was moderately reduced (43.2%; normal 70%–120%), vWF:Ag was markedly elevated (493%; normal 61.3–157.8%), and ADAMTS13 inhibitor testing was negative. A Sequential Organ Failure Assessment (SOFA) score >2 was consistent with septic shock complicated by septic cardiomyopathy.

Sepsis bundle management was initiated immediately, including empiric broad-spectrum antimicrobial therapy, fluid resuscitation, vasopressor support, and close organ function monitoring. Endotracheal intubation and mechanical ventilation were required for respiratory failure. Bronchoscopy was performed, and bronchoalveolar lavage fluid and blood samples were obtained for microbiological analysis. Empiric meropenem, hydrocortisone (200 mg/day), and sivelestat (0.3 g/day) were administered. Intra-aortic balloon pump support and continuous renal replacement therapy were instituted for circulatory and renal support, respectively.

By POD3, the patient's hemodynamic status and metabolic parameters showed partial stabilization. Microbiological analysis of bronchoalveolar lavage fluid identified mecA-positive *Staphylococcus aureus* and *Citrobacter species*, prompting the addition of linezolid to the antimicrobial regimen. Despite systemic improvement, coagulation abnormalities progressed, with further PT prolongation (35.7 s), continued decline in D-dimer (0.82 µg/mL), worsening thrombocytopenia (45 × 10⁹/L), persistently elevated fibrinogen levels (6.58 g/L), rising vWF:Ag (548%), and further reduction in ADAMTS13 activity (32.8%), consistent with significant dysregulation of the vWF/ADAMTS13 axis. Formal coagulation assessment yielded a SIC score of 6 (>4), while the International Society on Thrombosis and Haemostasis (ISTH) DIC score was 4 (<5), indicating significant sepsis-associated coagulopathy without progression to overt disseminated intravascular coagulation. The timing of serial ADAMTS13 activity and vWF:Ag measurements is shown in Figure 1 together with the corresponding coagulation parameters.

**Figure 1:**
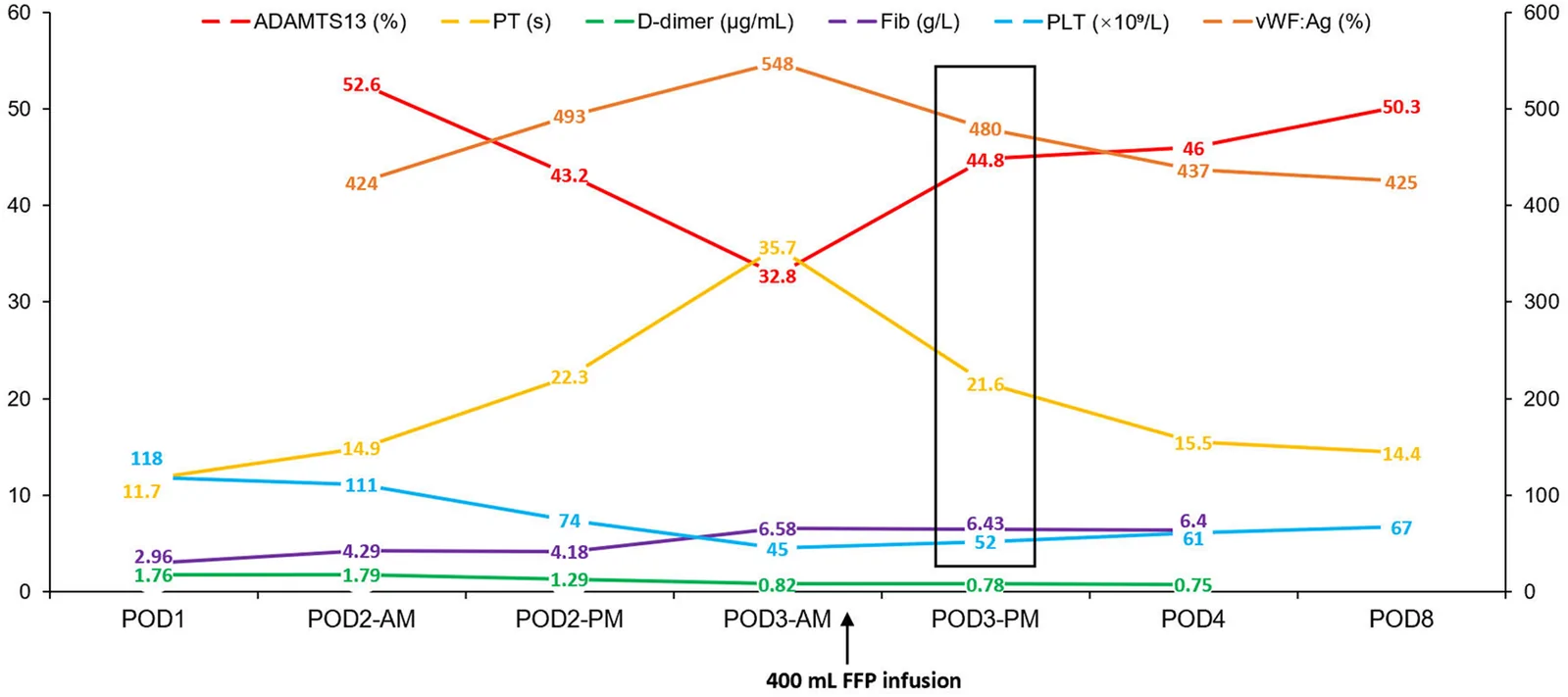
Dynamic changes in coagulation parameters and vWF/ADAMTS13 axis following cardiac surgery and sepsis. POD, postoperative day; AM, ante meridiem; PM, post meridiem; PT, prothrombin time; Fib, fibrinogen; PLT, platelet count; vWF:Ag, von Willebrand factor antigen; FFP, fresh frozen plasma.

In this context, 400 mL of fresh frozen plasma (FFP) was administered as adjunctive supportive therapy to supplement ADAMTS13 activity and address coagulation imbalance. Following plasma infusion, laboratory parameters improved temporally, including PT (21.6 s), platelet count (52 × 10⁹/L), vWF:Ag (480%), ADAMTS13 activity (44.8%), and stabilization of fibrinogen levels, while D-dimer remained low (0.78 µg/mL). Clinical and laboratory trends continued to improve thereafter (Table 1). Intra-aortic balloon pump support was discontinued on POD5, and the patient was discharged on POD16. At one-month follow-up, he demonstrated good functional recovery.

**Table 1:** Postoperative inflammatory and organ function parameters.

Parameters	POD1	POD2	POD3	POD5	POD8
Inflammatory markers					
IL-6 (pg/mL)	61.3	38,103.0	411.0	33.7	23.9
PCT (ng/mL)	0.59	1.39	47.28	26.22	1.85
WBC (×10⁹/L)	14.6	3.3	9.6	9.7	6.3
Organ function biomarkers					
hs-cTnI (ng/mL)	12.956	8.525	13.126	2.265	0.896
Scr (*μ*mol/L)	147	242	221	/	134

POD, Postoperative day; IL-6, Interleukin-6; PCT, Procalcitonin; WBC, White Blood Cell; hs-cTnI, high-sensitivity cardiac troponin I; Scr, Serum creatinine.

## Discussion

This patient developed severe sepsis and multiple organ dysfunction following cardiac surgery, accompanied by thrombocytopenia, schistocytes (2.6%), and laboratory evidence of endothelial and coagulation dysregulation. Given the overlap among TTP, DIC, SIC, and sepsis-associated TMA-like presentations in critically ill patients, a structured diagnostic assessment was essential. The three principal diagnostic considerations were differentiated as follows:

- Classic immune-mediated TTP: Although thrombocytopenia, microangiopathic hemolytic anemia, and organ dysfunction raised this possibility, several findings argued against classic TTP. ADAMTS13 activity was only moderately reduced, with a nadir of 32.8%, rather than severely deficient; ADAMTS13 inhibitor testing was negative; and the patient improved without therapeutic plasma exchange, which is generally required in immune-mediated TTP (6, 7).
- Overt DIC: The patient fulfilled criteria for SIC, with a score of 6 (>4) based on thrombocytopenia, prolonged PT, and organ dysfunction. However, the ISTH DIC score was 4 (<5), below the threshold for overt DIC. In addition, fibrinogen concentrations remained elevated rather than decreased, and D-dimer levels declined during the period of worsening thrombocytopenia. These features were not typical of fibrin-driven consumptive coagulopathy and therefore did not support overt DIC (8–10).
- Sepsis-associated TMA-like syndrome: This interpretation best accounted for the combined findings of microangiopathic hemolytic anemia, platelet-predominant thrombocytopenia, marked vWF elevation, relative ADAMTS13 reduction, and negative ADAMTS13 inhibitor testing, while criteria for classic TTP and overt DIC were not met. In the setting of severe sepsis, this profile suggests an endothelial injury- and vWF-dominant microthrombotic process, consistent with previously described endotheliopathy-associated “TTP-like” or sepsis-associated TMA-like syndromes (2, 11, 12).

It should be emphasized that 'sepsis-associated TMA-like syndrome' is used here as a descriptive term and does not imply equivalence to classic immune-mediated TTP.

The observed laboratory profile aligns with the “two-activation theory of the endothelium,” in which inflammatory and complement-mediated endothelial injury leads to excessive release of ultra-large vWF multimers (5, 13). In this framework, relative rather than absolute ADAMTS13 insufficiency may be sufficient to permit accumulation of highly prothrombotic vWF multimers, thereby promoting platelet adhesion and microvascular thrombosis. This mechanism offers a plausible explanation for the presence of schistocytes, consumptive thrombocytopenia, and organ dysfunction despite preserved or elevated fibrinogen levels.

The D-dimer trend was particularly informative in differentiating the suspected sepsis-associated TMA-like syndrome from overt DIC. D-dimer reflects fibrin formation followed by fibrinolytic degradation and is usually increased in conditions characterized by systemic fibrin-rich clot formation, such as overt DIC or venous thromboembolism (8, 10). In this patient, however, D-dimer levels declined from 1.29 to 0.82 µg/mL during the same period in which thrombocytopenia worsened, PT became more prolonged, and fibrinogen concentrations remained elevated. This pattern was not typical of progressive fibrin-driven consumptive coagulopathy.

Importantly, a falling or only mildly elevated D-dimer level does not exclude ongoing microvascular thrombosis when the thrombotic process is mainly platelet- and vWF-mediated. In sepsis-associated TMA-like syndrome, microthrombi are relatively enriched in platelets and vWF rather than fibrin, and therefore may generate less fibrin degradation product than classic DIC (9). Thus, in the present case, the declining D-dimer level should not be interpreted as evidence against microthrombosis. Instead, when considered together with schistocytes, platelet-predominant thrombocytopenia, markedly elevated vWF:Ag, relative ADAMTS13 reduction, preserved or increased fibrinogen, and an ISTH DIC score below the threshold for overt DIC, the D-dimer trend was more compatible with sepsis-associated TMA-like syndrome than with fibrin-driven coagulopathy.

Management focused primarily on rapid sepsis control and organ support, including antimicrobial therapy, mechanical ventilation, vasoactive support, intra-aortic balloon pump assistance, and continuous renal replacement therapy. FFP (400 mL) was administered as adjunctive supportive care in the setting of coagulation abnormalities and vWF/ADAMTS13 imbalance. Therapeutic plasma exchange was considered but not pursued because the patient did not meet diagnostic criteria for classic immune-mediated TTP, including severe ADAMTS13 deficiency or detectable ADAMTS13 inhibitors. In addition, specific evidence supporting TPE for sepsis-associated TMA-like syndrome remains sparse. Although a recent real-world study of TTP and TTP-like syndrome reported that infection was a common precipitating condition in the TTP-like group, the apparent benefit of plasma exchange was much less evident in TTP-like syndrome than in TTP (14). Therefore, TPE was not used as targeted therapy for sepsis-associated TMA-like syndrome in this case.

Although several laboratory parameters improved after FFP administration, any specific therapeutic benefit from FFP in this case remains speculative. The improvement occurred in the context of multiple concurrent interventions, including effective antimicrobial therapy, hemodynamic support, CRRT, IABP assistance, and the gradual resolution of sepsis-related endothelial dysfunction. Therefore, the clinical course should not be interpreted as evidence that plasma infusion directly improved sepsis-associated TMA-like syndrome. At most, FFP may have served as a nonspecific supportive measure, and no causal relationship between FFP administration and recovery can be established from this single case.

Finally, it should be acknowledged that sepsis-associated TMA and vWF-dominant microthrombosis remain areas of ongoing investigation and debate. Alternative interpretations of this case include severe sepsis-related endothelial activation without definitive TMA, underscoring the need for further prospective studies integrating dynamic endothelial and coagulation biomarkers.

## Conclusions

This case illustrates that postoperative sepsis following cardiac surgery may present as sepsis-associated TMA-like syndrome, which is distinct from classic TTP and overt DIC. The presence of microangiopathic hemolysis, platelet-predominant thrombocytopenia, and vWF/ADAMTS13 imbalance in the absence of fibrin-driven coagulopathy underscores the importance of structured diagnostic differentiation in critically ill patients. Adjunctive plasma therapy may be considered as part of individualized supportive care, although its specific role in this condition remains uncertain.

## Data Availability

The original contributions presented in the study are included in the article/Supplementary Material, further inquiries can be directed to the corresponding author.
